# Impact of Rheumatic Diseases on Oral Health-Related Quality of Life

**DOI:** 10.7759/cureus.32268

**Published:** 2022-12-06

**Authors:** Ashwag Y Aloyouny, Fatimah Almufarji, Ghadeer G Almutairi, Shahad Alkait, Maha Ali Al-Mohaya, Rasha Alserwi

**Affiliations:** 1 Basic Dental Science Department, Princess Nourah Bint Abdulrahman University, Riyadh, SAU; 2 Oral Medicine and Special Care Dentistry Department, Prince Sultan Military Medical City, Riyadh, SAU

**Keywords:** temporomandibular joint, sjogren syndrome, systemic lupus erythematosus, behcet's disease, rheumatic disease, oral function, oral manifestation, rheumatic arthritis, quality of life, oral health

## Abstract

Introduction

Oral-health-related quality of life (OHRQoL) represents a part of the general health-related quality of life (HRQoL). This OHRQoL indicates someone's subjective knowledge of a patient's oral health status, which is mostly associated with physical conditions and general HRQoL issues. A report by the World Health Organization labeled rheumatic and musculoskeletal diseases as the second most reported cause of disability worldwide. Considering their potential influence on the masticatory system, rheumatic diseases (RDs) can significantly affect oral health and the quality of life.

Objective

This study aimed to evaluate the impact of RDs on OHRQoL, including oral complaints, oral habits, oral functions, and dental care.

Materials and methods

This cross-sectional, multicenter study was conducted in multi-governmental medical and dental institutions in Riyadh, Saudi Arabia. The research was approved by the ethics committee Institutional Review Board (IRB). The validated surveys were distributed to the subjects manually. Data were analyzed using Statistical Package for the Social Sciences (SPSS) 23.0 software, and all parameters were expressed in frequencies and percentages.

Results

The study included 108 patients: 10 males (9.3%) and 98 (90.7%) females. Approximately 81% of the study group reported occasional changes in the quality of life caused by oral or temporomandibular joint (TMJ) problems. Dental caries and periodontal diseases were the most commonly reported complaints (73.1%). Regarding oral manifestations of RDs affecting the quality of life, 91.7% of participants never experienced difficulty speaking and taste changes; pain and discomfort in the mouth were always present in 4.6% of the participants.

Conclusion

Patients with RDs exhibited reduced OHRQoL, with several differences between the entities. Specifically, OHRQoL decreased in diseases with more oral manifestations, such as systemic lupus erythematosus (SLE) and rheumatoid arthritis (RA), which showed a high percentage in this study (SLE, about 27.8%; RA, 62%).

## Introduction

The World Health Organization defines quality of life as an individual's perception of their position in life in the context of the culture and value systems in which they live about their goals, expectations, standards, and concerns. Quality of life is a comprehensive concept in which the oral-health-related quality of life (OHRQoL) represents a part of the general health-related quality of life (HRQoL) [1]. OHRQoL indicates the subjective evaluation of the oral health status of a person and is primarily associated with physical conditions and general HRQoL issues. A report by the World Health Organization labeled rheumatic and musculoskeletal diseases as the second most reported cause of disability worldwide [2]. Rheumatic diseases (RDs) affect joints, muscles, ligaments, tendons, bones, and connective tissues. They constitute a heterogeneous group of disorders that cause high morbidity in the affected patient [1]. Patients with RDs predominantly suffer from at least one or more comorbid conditions associated with persistent inflammatory activity or disease-related organ damage [3]. Some types of anti-rheumatic drugs (ARDs), such as biological drugs, may lead to medication-related osteonecrosis of the jaw and affect the OHRQoL [1]. These comorbidities may be related to RDs or their treatment or be completely independent [3]. Considering the role of the masticatory system in speech, mastication, taste perception, eating, appearance, and self-confidence, RDs significantly impact oral health [4].

ARDs often produce multiple oral manifestations, such as oral aphthous ulcers, xerostomia, strawberry-like gingiva, microstomia, microcheilia, periodontitis, and tooth loss. These symptoms may interfere with the patient's daily activities and quality of life [4]. Multiple side effects of ARDs may also interfere with patients' OHRQoL [5]. Therefore, this study aimed to evaluate the impact of oral lesions on the quality of life among patients with RDs. This study aimed to evaluate the impact of RDs on OHRQoL, including oral complaints, oral habits, oral functions, and dental care.

## Materials and methods

Participant selection

This cross-sectional, multicenter study was conducted in multi-governmental medical and dental institutions in Riyadh, Saudi Arabia, from July 2020 to November 2021. The participants were selected using a simple random sampling procedure.

A total of 108 individuals recruited from rheumatology clinics and diagnosed with at least one rheumatological disease participated in the study. Data on clinical manifestations were collected from the patient's history and gleaned via a questionnaire. Female and male patients, age ≥14 years, literate, capable of completing the study questionnaire, and meeting the classification criteria of one of the following diseases: systemic lupus erythematosus (SLE), primary Sjogren syndrome (SS), fibromyalgia syndrome (FMS), rheumatoid arthritis (RA), Behcet's disease (BD), spondyloarthritis (SpA), systemic sclerosis (SSc), and idiopathic inflammatory myopathies (IIM) were included. The study excluded human immunodeficiency virus, hepatitis C, and hepatitis B infections and patients diagnosed with severe psychiatric disorders. Patients with metabolic conditions, such as diabetes and hyperthyroidism, were also excluded owing to their strong links to oral symptoms.

Study design

Each participant signed the Institutional Review Board (IRB) approved informed consent form authorized by the ethics committee of the collaborating centers and Princess Nourah Bint Abdulrahman Institutional Review Board with approval number 22-0464. Skilled dentists and oral medicine experts conducted oral assessments by close inspection and palpation to detect any mucosal lesions, growth, pigmentations, and any abnormality in the temporomandibular joint (TMJ) areas. The subjects were given a verified questionnaire and fulfilled it manually. Each participant was personally interviewed to review the questionnaire. The data obtained from the questionnaire consisted of the participant's demographic information, including age, gender, race, occupation, educational level, marital status, pregnancy for females, and complete medical, dental, and family history.

The questionnaire survey also included the history of RD, the presence of oral lesions or complaints or malfunction, and OHRQoL. The Oral Health Impact Profile (OHIP) included questions that measured the effect of oral conditions on the quality of life using a 3-point Likert scale (always, sometimes, and never), a gold standard measure for OHRQoL to assess functional, social, and psychological outcomes of oral conditions.

Following data collection, a Chi-square test was used to compare participant groups based on gender, age-range and job title. The threshold for significance was defined as ≤ p 0.05. Statistical Package for the Social Sciences (SPSS) 23.0 was used to collect, organize, and statistically analyze the data for Windows (SPSS, Inc., Chicago, IL, USA). Microsoft Excel was used to create representative graphs. The STrengthening the Reporting of OBservational studies in Epidemiology (STROBE) criteria were followed in our study.

## Results

**Figure 1:**
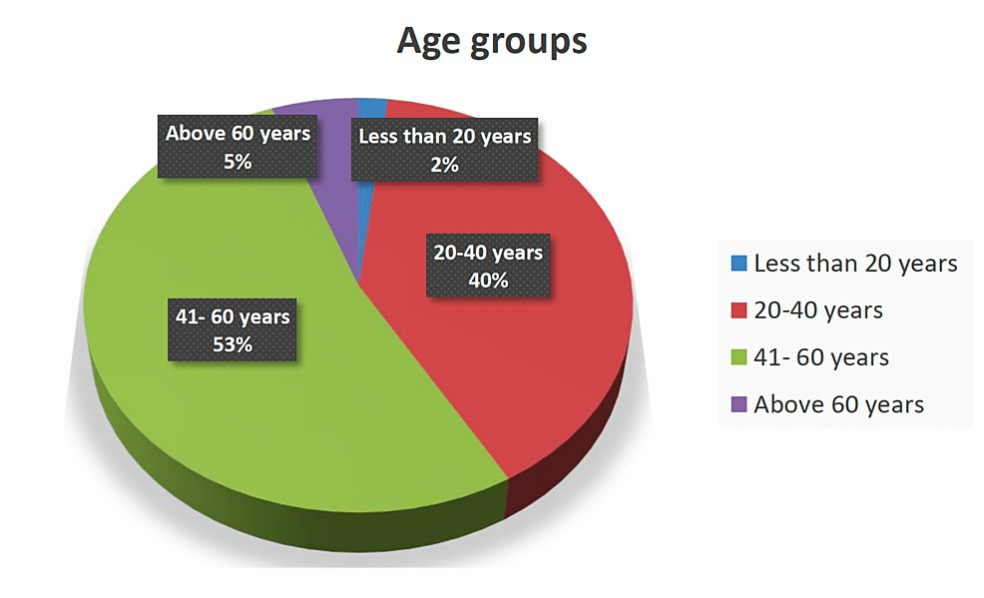
Pie chart illustrating age groups of the study participants

The study included 108 patients (10 males and 98 females). The mean age of the participants was 42 ± 12 years, ranging from 14 to 70 years (Figure 1). Most participants were Saudi citizens (predominantly females and homemakers). Around 69.4% of the patients were married, 24.1% were single, and 6.5% were widowed. None of the women participants were pregnant. Unquestionably, RA, which affected 62% of the patients, was the most prevalent illness. Prevalence of SpA, SLE, FMS, and SS was 2.8%, 27.8%, 5.6%, and 2.8%, respectively. About 5% of the subjects had additional illnesses, including psoriasis, Kikuchi disease, Henoch-Schoenlein purpura, Still's disease, and perinuclear anti-neutrophil cytoplasmic antibody vasculitis. Notably, six of the 108 participants had multiple RDs. Disease duration since diagnosis ranged from one month to 26 years (mean: 6.6 ± 5.7 years).

**Figure 2:**
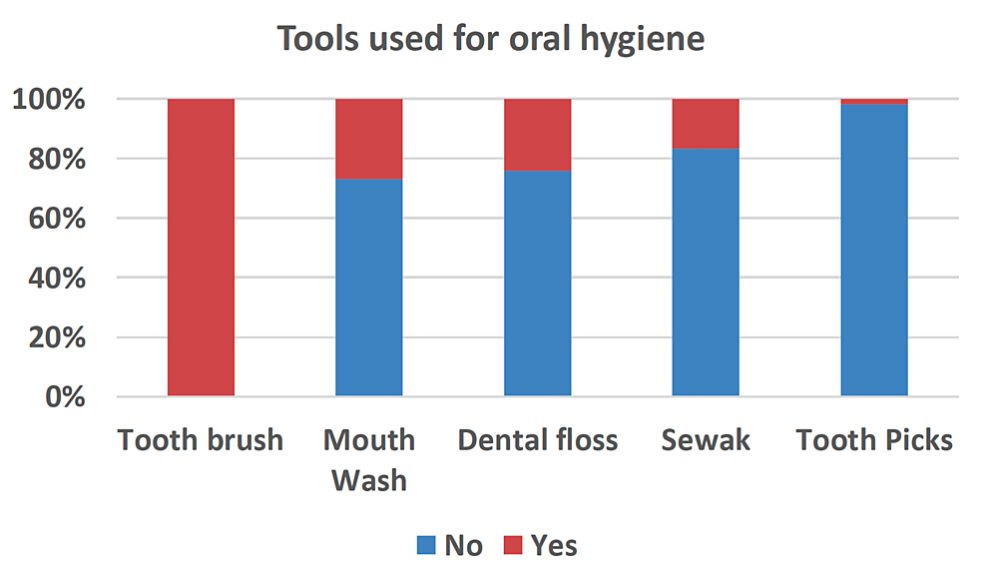
Bar chart representing the participants' different oral hygiene tools.

The participants used different tools for oral hygiene, including toothbrushes, mouthwash, dental floss, Sewak, and toothpicks. As hypothesized, the toothbrush was the most frequently used tool, and only 25% of participants reported using mouthwash and dental floss. A small percentage of the participants reported using Sewak, and less than 2% used toothpicks (Figure 2). About 60% of the participants had good dental habits and oral hygiene; they brushed their teeth two to three times daily; 32.4% brushed once daily, 2.8% brushed every three days, and 5.6% brushed every week.

Oral manifestations of autoimmune RDs impacted the quality of life in the following ways: 91.7% of participants never had difficulty speaking or altered taste sensations. In 4.6% of cases, pain and discomfort in the mouth were always present. 1.9% of patients had chewing difficulty on a regular basis. Furthermore, a small percentage, about 2%, always struggled with mouth cleaning. Meal interruptions caused by oral or TMJ problems sometimes occurred in 18.5% of cases. Almost one-third of the participants felt embarrassed occasionally due to oral or TMJ issues. Nearly half of the patients had difficulty with everyday tasks or could not function due to oral or TMJ problems. More than half of the participants were occasionally dissatisfied with their overall quality of life. Approximately 81% of the study participants often reported changes in their quality of life due to oral or TMJ problems (Table 1).

**Table 1:** Presents the oral manifestations of rheumatic diseases affecting the quality of life.

Question	Always		Sometimes		Never	
	No.	%	No.	%	No.	%
Difficulty speaking	0	0	9	8.3	99	91.7
Taste changes	0	0	9	8.3	99	91.7
Pain and discomfort in the mouth	5	4.6	44	40.8	59	54.6
Difficulty chewing	2	1.9	36	33.3	70	64.8
Difficulty in mouth cleaning	2	1.9	44	40.7	62	57.4
Interruption of meals due to oral or TMJ problems	1	0.9	20	18.5	87	80.6
Embarrassment due to oral or TMJ problems	3	2.8	32	29.6	73	67.6
Difficulty in daily functional routine due to oral or TMJ problems	4	3.7	56	51.9	48	44.4
Less satisfaction with your quality of life	5	4.6	60	55.6	43	39.8
Inability to function due to oral or TMJ problems	1	0.9	50	46.3	57	52.8
Change in quality of life caused by oral or TMJ problems in rheumatic diseases	16	14.8	87	80.6	5	4.6

Dental caries and periodontal diseases were the most reported oral complaints among the study group (73.1%). TMJ clicking and xerostomia affected nearly half of the study participants. Other oral complaints included pain during mouth opening, TMJ muscle pain, difficulty swallowing, oral ulcers, red or white macules, and angular cheilitis (Table 2).

**Table 2:** The oral complaints among the study participants.

Question	Response	No.	%	Question	Response	No.	%
Xerostomia	No	57	52.8%	Oral ulcers	No	95	88.0%
	Yes	51	47.2%		Yes	13	12.0%
Difficulty swallowing	No	99	91.7%	Pain on mouth opening	No	77	71.3%
	Yes	9	8.3%		Yes	31	28.7%
Dental caries	No	29	26.9%	Difficulty with mouth opening	No	95	88.0%
	Yes	79	73.1%		Yes	13	12.0%
Periodontal disease	No	29	26.9%	Clicking on mouth opening	No	56	51.9%
	Yes	79	73.1%		Yes	52	48.1%
Red/white macules	No	98	90.7%	Pain in muscles around TMJ	No	90	83.3%
	Yes	10	9.3%		Yes	18	16.7%
Angular cheilitis	No	101	93.5%				
	Yes	7	6.5%				

Approximately 57% of the study group reported no difficulty scheduling dental appointments, while less than one-third reported the opposite. Almost 14% of participants stated that oral medicine was not always available in dental centers. Around 36% of participants believed that a general dentist could treat them effectively, while about 26% were dissatisfied with general dentists' knowledge and skills in managing patients with RA. Only six patients (5.6%) reported rejection from dental care at a private clinic. Almost two-thirds of those polled were pleased with their dental care, while 8.4% were dissatisfied. In 25% of the patients, the time elapsed between the diagnosis of autoimmune RD and the first dental visit was one to three years. Regarding payment for dental visits, about 54% had no problems, while in a small group, about 14% always had difficulty with dental expenses (Table 3).

**Table 3:** The study participants' dental care.

Question	Response	No.	%
Difficulties in scheduling dental visits	Difficulty in appointment	31	28.7
	Oral medicine specialist not available	15	13.9
	No difficulty	62	57.4
Do you think a general dentist can treat you	No	28	26
	Yes	39	36
	Don’t know	41	38
Have you been rejected to get private dental treatment	No	102	94
	Yes	6	5.6
Are you satisfied with the received dental care?	Satisfied	75	69.5
	Neutral	24	22.2
	Not satisfied	9	8.4
Time elapsed between rheumatic disease diagnosis and first dental visits	0-6 months	40	37
	6-12 months	31	28.7
	1-3 years	27	25
	3-6 years	8	7.4
	More than 6 years	2	1.9
Difficulty in dental payment	Always	15	13.9
	Sometimes	35	32.4
	Never	58	53.7

As shown in Table 4, a comparison of oral functions and quality of life by age group revealed statistically significant results on difficulty speaking, pain and oral discomfort, interruption of meals, and inability to function and work due to dental or TMJ problems.

**Table 4:** Comparing the oral functions and the quality of life according to age group. *: statistical significance (P ≤ 0.05)

Question	Age group	Always		Sometimes		Never		P-value
		No.	%	No.	%	No.	%	
Difficulty speaking	<20	0	0	0	0	2	100	0.03*
	21-40	0	0	5	11.6	38	88.4	
	41- 60	0	0	2	3.5	55	96.5	
	> 60	0	0	2	33.3	4	66.7	
Taste changes	<20	0	0	0	0	2	100	0.13
	21-40	0	0	2	4.7	41	95.3	
	41- 60	0	0	5	8.8	52	91.2	
	> 60	0	0	2	33.3	4	66.7	
Pain/Discomfort in mouth	<20	0	0	0	0	2	100	0.04*
	21-40	3	7	20	46.5	20	46.5	
	41- 60	0	0	23	40.4	34	59.6	
	> 60	2	33.3	1	16.7	3	50	
Difficulty chewing	<20	0	0	0	0	2	100	0.36
	21-40	1	2.3	14	32.6	28	65.1	
	41- 60	0	0	20	35.1	37	64.9	
	> 60	1	16.7	2	33.3	3	50	
Difficulty in mouth cleaning	<20	0	0	1	50	1	50	0.65
	21-40	1	2.3	14	32.6	28	65.1	
	41- 60	1	1.8	24	42.1	32	56.1	
	> 60	0	0	5	83.3	1	16.7	
Interruption of meals due to teeth or TMJ problems	<20	0	0	0	0	2	100	0.00*
	21-40	1	2.3	7	16.3	35	81.4	
	41- 60	0	0	10	17.5	47	82.5	
	> 60	0	0	3	50	3	50	
Embarrassment due to oral or TMJ problems	<20	0	0	1	50	1	50	0.10
	21-40	2	4.7	12	27.9	29	67.4	
	41- 60	1	1.8	16	28	40	70.2	
	> 60	0	0	3	50	3	50	
Difficulty in daily functional routine due to teeth or TMJ problems	<20	0	0	2	100	0	0	0.06
	21-40	2	4.6	18	41.9	23	53.5	
	41- 60	1	1.8	32	56.1	24	42.1	
	> 60	1	16.7	4	66.6	1	16.7	
Inability to function and work due to teeth or TMJ problems	<20	0	0	2	100	0	0	0.03*
	21-40	0	0	16	37.3	27	62.8	
	41- 60	0	0	28	49.2	29	50.9	
	> 60	1	16.7	4	66.7	1	16.7	
Less satisfaction with quality of life	<20	0	0	2	100	0	0	0.46
	21-40	3	7	21	48.8	19	44.2	
	41- 60	1	1.8	33	57.9	23	40.3	
	> 60	1	16.7	4	66.6	1	16.7	

Furthermore, 30% of male and 25.5% of female participants felt that a general dentist could not treat a patient with autoimmune RDs, whereas almost 40% of females believed that a general dentist could treat them (a statistical significance of 0.03). Many participants reported payment difficulties; 15.3% of females always had problems. In addition, 40% of males sometimes had concerns about dental charges compared to 31.7% of females. In contrast, 60% of males and 53% of females never had difficulty paying for dental care, with a P-value of 0.04 (Table 5).

**Table 5:** Comparison of dental care according to gender. *: statistical significance (P ≤ 0.05)

Question	Response	Male		Female		P-value
		No.	%	No.	%	
Difficulties in dental visits	Difficulty in appointment	3	30.0	28	28.6	0.93
	Oral medicine specialist not available	1	10	14	14.3	
	No difficulty	6	60	56	57.1	
Do you think a general dentist can treat you	No	3	30	25	25.5	0.03*
	Yes	0	0	39	39.8	
	Do not know	7	70	34	34.7	
Have you been rejected to get private dental treatment	No	10	100	92	93.9	0.42
	Yes	0	0	6	6.1	
Are you satisfied with the received dental care?	Satisfied	8	80	67	68.4	0.84
	Neutral	2	20	22	22.4	
	Not satisfied	0	0	9	9.2	
Time elapsed between rheumatic disease diagnosis and first dental visits	0-6 months	7	70	33	33.7	0.22
	6-12 months	1	10	30	30.6	
	1-3 years	2	20	25	25.5	
	3-6 years	0	0	8	8.2	
	More than 6 years	0	0	2	2	
Difficulty in dental payment	Always	0	0	15	15.3	0.04*
	Sometimes	4	40	31	31.7	
	Never	6	60	52	53	

## Discussion

Autoimmune RDs are a heterogeneous group of disorders that cause high morbidity in affected patients. Thus, despite their rarity, their clinical relevance is apparent. There has been incredible progress in treating RDs in recent decades, but there is one focus on the care of the quality of life of rheumatic patients. Although quality of life represents a broad concept, OHRQoL describes a patient's subjective perception of oral health status. It is primarily related to physical conditions of the mouth but also to HRQoL issues. Therefore, in every dimension of OHRQoL, whether psychosocial or functional, patient-reported outcomes differ by response format, the number of items, or the context of use [1]. The present study determined the health-associated quality of life, focusing more on the OHRQoL.

Out of 108 patients, the majority were females (90.7%). Due to both exogenous and endogenous hormonal changes, genetic differences, and lifestyles, women are more susceptible to autoimmune RDs [6]. The most common disease was RA, affecting 62% of the patients; SLE affected 27.8% of the participants. The sample size showed that most patients maintained good oral hygiene by brushing two to three times daily. Goma et al. reported that patients with RA had reduced quality of life in several aspects, such as level of independence, physical health, environment, and personal beliefs, compared to the healthy population [7]. This finding explained the difficulty sometimes in mouth cleaning in about 41% of the patients. A high percentage of the study participants had caries and periodontal disease, which are the main causative factors of tooth loss if untreated [8]. Teeth loss has a definitive impact on patient satisfaction with appearance, general performance, and eating; the level of satisfaction with dentition and daily living decreases with an increased number of missing teeth [9]. A study describing the results of a systematic review of oral function found that disability and performance of mastication were linked to the number of teeth [10]. One study revealed a more significant loss of periodontal attachment and alveolar bone in early-stage RA, implying that patients should receive intensive dental care to limit periodontal damage, especially in the early stages [5]. Another study showed that improvements in oral hygiene and early periodontal therapy could reduce the severity of systemic conditions [11].

An evaluation of patient responses and oral manifestations revealed that many patients complained of TMJ problems that resulted in impaired oral function, change in the quality of life, and clicking on mouth opening; these findings were consistent with different studies. Bessa-Nogueira et al. showed that out of 61 patients, 49.2% had noisy TMJ sounds, 19.7% had TMJ clicking, and about 40% had TMJ pain on palpation. Also, patients with RA are usually at risk of TMJ disorders as the disease is characterized by synovitis [12].

TMJ complaints were attributed to chronic inflammation that affects the joints, causing pain and structural abnormalities. A study on 32 patients with pain in the TMJ or jaw muscles reported that the symptoms varied from constant severe pain and trismus to infrequent discomfort and pain with insignificantly impaired function [13]. Toothache and severe TMJ pain can hamper a patient's daily work and activities, especially if the patient has a low pain threshold. Patients with autoimmune diseases suffer from oral complaints, such as hyposalivation, dental caries, and periodontal diseases, which also significantly impact the patients' OHRQoL. These dental problems could be attributed to the underlying mechanism of RA or side effects of medications, such as non-steroidal anti-inflammatory drugs and disease-modifying ARDs [5].

This study showed no significant difference in taste perception, difficulty in chewing, difficulty in mouth cleaning, embarrassment due to TMJ problems, difficulty in everyday tasks, less satisfaction with the quality of life, and change in the quality of life. Another study showed that an increase in RA severity led to an increased impairment of some oral activities, such as eating hard food, chewing, and yawning [13]. The difference between age groups was statistically significant for difficulty speaking, pain, oral discomfort, interruption of meals, and increasing incidence with age. Similarly, one study showed that most RA patients had a poor quality of oral health, and the deterioration of disease and aging decreased the General Oral Health Assessment Index [4]. In our study, participants reported oral dysfunction according to the age group, including difficulty speaking, pain and discomfort, interruption of meals due to dental and TMJ problems, and inability to function and work, which showed statistically significant values. Most participants had no interruptions in their meals. Many patients complained of pain and oral discomfort in different age groups. Most of them were in the age group of 21 to 40 years.

Most study participants reported no difficulties with dental visits; however, 28.7% had problems, and 13.9% said that an oral medicine specialist was not always available.

In this study, only 36% considered that a general dentist could treat them well. General dentists are trained to manage patients with RD and identify the signs and symptoms of the disease. However, patients with RD sometimes require special considerations in treating oral lesions. Moreover, biopsy procedures may be needed to confirm the diagnosis of some diseases, such as suspected oral lichen planus [14]. Therefore, it is sometimes essential to refer the patients to an oral medicine specialist for adequate disease management.

Only six patients (5.6%) reported rejection from dental treatment in a private clinic. Some dentists might refuse to treat patients with systemic conditions due to a lack of knowledge and awareness of the disease. Moreover, some private clinics may lack the facilities in case of an emergency in the dental chair, thus creating treatment challenges. Bindakhil et al. mentioned that one barrier to diagnosing and treating oral and maxillofacial disorders was the absence of qualified and trained physicians [15].

Most participants were satisfied with the dental care they received in a governmental or private clinic or by a specialist or general dentist. Dental treatment should be initiated immediately after diagnosis of RD for a better prognosis. In some cases, dentists could be the first to diagnose rheumatic illness since it often produces oral lesions in the early stages, which may be detected in dental examinations [16].

In this study, after the established diagnosis of one of the RDs, almost one-third of the participants had their first dental visit in less than six months, the other one-third had a six to 12 months delay, and the last group delayed the dental treatment by one to three years.

Regarding the payment for dental visits, half the participants never had difficulty paying for dental treatment, probably because most participants received dental treatment at governmental clinics. A study conducted in Saudi Arabia on the barriers to access and utilization of dental services reported that some obstacles to obtaining dental services were no dental insurance (64.5%) and unaffordable price (61.3%) [17]. Another study reported cost (48.7%) as a barrier to accessing dental care, cited by caregivers on behalf of their patients [18].

The limitations of this study were the small sample size due to the limited included centers, and it was conducted only in Riyadh, Saudi Arabia.

## Conclusions

Patients with RDs exhibit reduced OHRQoL, with several differences between the entities, especially in conditions with more oral manifestations, such as SLE and RA, which showed a high percentage of about 27.8% and 62%, respectively, in the present study. In addition to the OHRQL, HRQoL was affected by disease-related parameters and ARDs. Therefore, patients with RDs should receive multidisciplinary dental care. Future studies should use a standardized methodology to measure general and disease-specific OHRQoL. Further research should focus on the effects of ARDs in improving the quality of life of RD patients.
